# Performance evaluation of a new automated skin flash method for radiotherapy of breast cancer

**DOI:** 10.1016/j.phro.2025.100810

**Published:** 2025-07-10

**Authors:** Topi Nykänen, Ville Raatikainen, Aarno Kärnä, Tuomas Koivumäki

**Affiliations:** a Department of Medical Physics, Hospital Nova of Central Finland, Wellbeing Services County of Central Finland, Jyväskylä, Finland; b Department of Physics, University of Jyväskylä (JYU), Survontie 9 C, 40014 Jyväskylä, Finland

**Keywords:** Radiotherapy, Volumetric modulated arc therapy, Breast cancer, Skin flash, Treatment planning

## Abstract

In volumetric modulated arc therapy of breast cancer, an outer margin for the target is needed. This study evaluated the clinical and planning target volume coverage and surface dose using an automated skin flash method compared to the virtual bolus method. Body outline changes of 4, 8 and 12 mm were simulated in 20 breast cancer patients. Median *V_95_* and *V_90_* clinical target volume coverage and surface dose remained at 90.1 %, 98.7 % and 85.7 % or above with the automated skin flash method. The median results of automated skin flash method were close to virtual bolus method.

## Highlights

• Performance of automatic skin flash for breast cancer radiotherapy was evaluated.
• Radiotherapy plans for virtual bolus and automatic skin flash were compared.
• Breast deformations were simulated by 4 mm, 8 mm and 12 mm expansions.
• Median results between both methods were close to each other.

## 1 Introduction

The dose distribution in breast cancer radiotherapy (RT) can be affected by tissue deformations, such as swelling, or positional changes during the treatment. Due to anatomical changes and motion during treatment as well as setup uncertainties, an outer margin is required in treatment planning to ensure adequate dose coverage at the skin surface [1].

Breast cancer RT has traditionally been implemented using tangential fields, employing techniques such as wedges, Field-in-Field (FinF) method or intensity-modulated radiotherapy (IMRT) [2,3]. In tangential techniques, the collimator is opened beyond the skin allowing dose to superficial regions, even with substantial soft tissue deformation. In recent years, volumetric modulated arc therapy (VMAT) has become more common, complementing or replacing conventional treatment techniques in breast cancer RT [[4], [5], [6], [7], [8], [9]]. VMAT offers advantages, including the ability to reduce radiation dose to critical structures such as the heart and ipsilateral lung, and reducing the risk of radiotherapy induced toxicities [10,11], while improving dose homogeneity in the planning target volume (PTV) [3,7,12]. However, the low dose volume in FinF and other tangential techniques is typically smaller than in VMAT plans [[13], [14], [15]]. With modern VMAT techniques, the expansion of the dose fluence beyond the body contour depends on the treatment planning system (TPS). If the TPS does not have a dedicated tool to expand the dose fluence outside of skin, a manual virtual bolus (VB) method has been introduced [6,8,[16], [17], [18], [19], [20], [21], [22]].

The aim of this study was to evaluate a new automated skin flash method compared to the virtual bolus method in terms of CTV and PTV coverage and mean skin dose with simulated swelling.

## 2 Materials and methods

This retrospective study included 20 patients with left-sided breast cancer. The study was approved by the Wellbeing Services County of Central Finland. All patients were originally imaged and treated using deep inspiration breath-hold (DIBH) in supine position on Extended Wing Board with U-grip Handle (CQ Medical, USA), with their arms positioned above the head. Planning CT scans were acquired with Somatom Confidence (Siemens Healthcare GmbH, Germany) with a slice thickness of 2 mm.

Each patient was prescribed a total dose of 40.05 Gy in 15 fractions. Clinical target volume (CTV) was delineated by a radiation oncologist following the ESTRO guidelines [23]. PTV was created by adding a 5 mm margin to the CTV. The patient cohort consisted of 10 whole breast cases and 10 cases with nodal involvement. The median CTV volume was 1076 cm^3^ [492–3002 cm^3^] and the median PTV volume was 1314 cm^3^ [716–3476 cm^3^].

Four VMAT plans were created for each patient. One plan used the conventional virtual bolus (VB) method and RapidArc (Varian Medical Systems, USA), and the other three utilized the new automated skin flash (ASF) feature with RapidArc Dynamic. The plans were optimized for a TrueBeam (Varian Medical Systems, USA) linear accelerator equipped with a Millenium 120 MLC, using a beam energy of 6 MV. The plans were optimized in Eclipse (v 18.1) using the Photon Optimizer algorithm. Dose calculation was performed using Acuros XB algorithm (v 18.1) with a dose matrix resolution of 2.5 mm. All plans were optimized according to dose objectives presented in Supplementary Table S1.

The VB plan consisted of four arcs, with two anterior and two posterior partial arcs (Supplementary Fig. S1). Two ASF plans used four arcs, similar to the VB plan, and one was optimized with two arcs. The isocenter was located at the interface between the ipsilateral lung and the chest wall, at the middle axis of the PTV. All plans included split arcs, either with or without a gap between the anterior and posterior arcs. The anterior arcs ranged from approximately 290° [285°–296°] to 45° [30°–60°], and the posterior arcs ranged from 75° [60°– 80°] to 160° or 179°. The static collimator angles ranged from 5° to 25°, and from 335° to 355° in anterior and posterior arcs, respectively. ASF configurations used one (four arc plans) or four (two arc plans) tangential static angle modulated ports (STAMP) per arc, with an 8° or 10° gap between each STAMP. One four arc ASF plan set was optimized with the STAMP weight set to ‘Arc Dominant’ to closely mimic the VB plan. On the other two ASF plans the STAMP weight was set to ‘Balanced’. Four arc plans used a static collimator. The two arc plans used dynamic collimator which was set to optimize between static angles. This fixes the collimator angle at each STAMP and the collimator rotates between STAMPs.

In the VB plans, the PTV was expanded outside the body contour by 13 mm, and 16 mm optimization bolus was applied using a Hounsfield Unit (HU) of –100. The final dose distribution was calculated without the optimization bolus. The plan was normalized to the mean dose of the PTV_skin_, which was defined as the PTV cropped by 3 mm from the skin. CTV_skin_ was created similarly by cropping the original CTV by 3 mm. The ASF plans were optimized with corresponding settings using a 13 mm PTV extension, and a virtual bolus HU value of –100. The ASF function similarly applies a 3 mm larger optimization bolus than the PTV extension. Unlike with the VB method, in the ASF method the optimization bolus density is dependent on the angle of beam incidence.

**Fig. 1:**
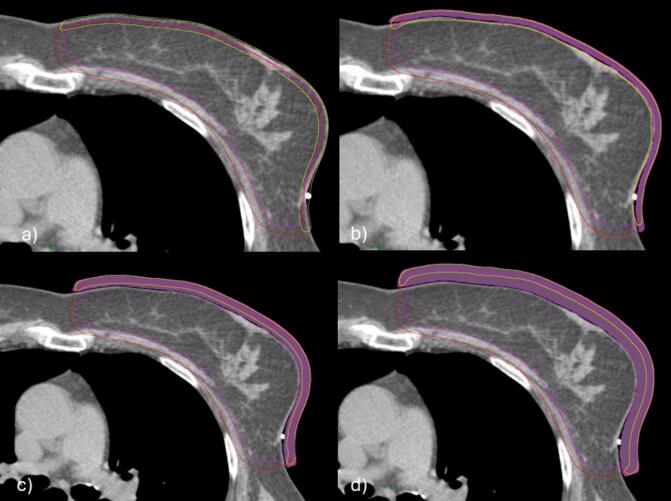
Original CT image (a) and expanded CTs with 4 mm (b), 8 mm (c) and 12 mm (d) of simulated swelling. Original and expanded CTV_skin_ (magenta) and PTV_skin_ (red) structures. Skin structure (yellow) was defined as the first 5 mm from the surface of the body. (For interpretation of the references to colour in this figure legend, the reader is referred to the web version of this article.)

For the evaluation, three modified CTs were created by extending the soft tissue anteriorly and laterally by 4, 8, and 12 mm outside the original body contour to simulate swelling (Fig. 1). The body contour was adjusted to mimic tissue swelling, and the CTV_skin_ and PTV_skin_ structures were expanded to match the new body contour. HU value of –100 was applied to the expanded body contour. The plans were recalculated using the original treatment plans in the modified structure sets. The recalculated plans were normalized to have the same monitor units as the original plans. CTV_skin_ and PTV_skin_ *V*_95_ (%) and *V*_90_ (%) coverages were collected. Additionally, a skin structure was defined as a volume of 5 mm from the surface of the body structure inside the PTV in each structure set. Mean dose in the skin structure (*D*_skin_) of each plan was recorded as percentage of planned dose (*D*_skin_ (%)) from the original plan and the expanded plans. Wilcoxon signed-rank test (IBM SPSS v29, USA) was used to evaluate the statistical significance (*p* < 0.05) between VB and ASF plans.

## 3 Result

All original plans achieved the PTV_skin_ *V*_95_ coverage of 95 % (Table 1). Homogeneity and conformity index results are presented in Supplementary materials.

**Table 1:** Median of CTV_skin_ and PTV_skin_ coverages and median skin doses (*D_skin_*) in virtual bolus (VB) and automated skin flash (ASF) treatment plans. Minimum and maximum values are presented in square brackets. (B = Balanced, AD = Arc Dominant).

	CTV_skin_		PTV_skin_		Skin
	*V*_95_ (%)	*V*_90_ (%)	*V*_95_ (%)	*V*_90_ (%)	*D_skin_* (%)
	Original				
**4 ARC VB**	98.4	100.0	95.7	99.7	91.3
	[96.8, 99.4]	[99.5, 100.0]	[95.0, 97,4]	[99.3, 99.9]	[89.4, 93.0]
**4 ARC AD ASF**	97.6	99.8	95.8	99.6	89.7
	[95.5, 98.7]†	[98.8, 100.0]†	[95.0, 97.1]	[98.7, 100.0]†	[84.9, 91.2]†
**4 ARC B ASF**	98.1	99.9	96.0	99.6	90.5
	[96.0, 99.3]‡*	[99.5, 100.0]‡	[95.0, 97.3]	[99.3, 99.9]	[88.8, 93.0]‡*
**2 ARC B ASF**	98.2	99.9	95.5	99.6	90.7
	[95.9, 99.3]‡*	[99.6,100.0]‡**	[95.1, 97.0]**	[99.3, 99.9]	[89.3, 93.4]‡*

	Expansion 4 mm				
**4 ARC VB**	96.5	99.7	94.1	99.5	88.6
	[94.9, 98.6]	[98.9, 100.0]	[92.0, 95.8]	[98.7, 99.8]	[86.4, 90.8]
**4 ARC AD ASF**	94.5	99.3	93.4	99.1	86.8
	[90.0, 97.0]†	[97.1, 99.9]†	[90.8, 95.2]†	[97.7, 99.7]†	[84.6, 88.7]†
**4 ARC B ASF**	95.0	99.5	93.7	99.3	87.5
	[92.7, 98.4]‡*	[98.5, 100.0]‡*	[92.3, 95.9]	[98.3, 99.8]‡*	[84.9, 90.4]‡*
**2 ARC B ASF**	95.3	99.6	93.7	99.3	87.8
	[93.5, 98.2]‡*	[98.6, 100.0]‡**	[92.5, 96.4]	[98.6, 99.8]‡*	[86.0, 91.4]‡*

	Expansion 8 mm				
**4 ARC VB**	95.5	99.6	92.4	99.2	87.2
	[93.2, 97.5]	[98.5, 99.9]	[89.1, 94.0]	[98.4, 99.7]	[85.4, 89.6]
**4 ARC AD ASF**	92.8	99.1	91.5	98.8	85.9
	[84.9, 96.4]†	[95.9, 99.8]†	[84.9, 94.1]†	[96.5, 99.5]†	[83.5, 88.2]†
**4 ARC B ASF**	93.4	99.2	91.7	98.8	86.3
	[88.0, 98.1]‡*	[97.4, 99.9]‡*	[87.2, 95.3]	[97.7, 99.7]‡*	[83.9, 89.8]‡*
**2 ARC B ASF**	94.1	99.3	91.6	99.0	86.7
	[89.6, 98.5]‡*	[98.2, 100.0]‡	[87.9, 96.4]	[98.0, 99.8]‡*	[84.2, 91.1]‡*

	Expansion 12 mm				
**4 ARC VB**	92.5	99.0	89.8	98.8	86.2
	[89.4, 95.3]	[98.3, 99.5]	[85.8, 92.2]	[98.2, 99.4]	[84.1, 88.0]
**4 ARC AD ASF**	90.1	98.7	88.6	98.6	85.7
	[76.9, 95.0]†	[96.6, 99.6]	[76.5, 92.9]	[96.6, 99.6]	[83.2, 87.9]
**4 ARC B ASF**	90.8	99.0	89.5	98.8	86.3
	[80.2, 97.1]‡	[97.4, 99.9]‡	[80.2, 95.0]‡	[97.8, 99.8]	[82.8, 90.1]‡
**2 ARC B ASF**	90.5	99.0	88.9	98.8	86.2
	[82.7, 97.9]‡*	[98.0, 99.9]‡	[81.9, 96.5]	[97.6, 99.8]	[84.7, 90.9]

† Denotes statistically significant difference between VB and arc dominant four arc ASF (*p < 0.05)*.

‡ Denotes statistically significant difference between arc dominant four arc ASF and balanced four arc ASF or balanced two arc ASF (*p < 0.05)*.

* Denotes statistically significant difference between VB and balanced four arc ASF or balanced two arc ASF (*p < 0.05)*.

** Denotes statistically significant difference between balanced four arc ASF and balanced two arc ASF (*p < 0.05)*.

The median CTV_skin_ *V_95_* coverage was higher with the VB method in the original CT and all the expanded CTs compared to the ASF method (Table 1). The median CTV_skin_ *V_95_* coverage were within 2.7 percentage points (pp) in the expanded plans. The median CTV_skin_ *V_95_* coverage remained above 90 % in the 12 mm expanded CT with both methods. PTV_skin_ *V_95_* coverage were within 0.3 pp in the original plans. On the 12 mm expanded CT the arc dominant ASF plan PTV_skin_ *V_95_* coverage was lower by 1.2 pp than the VB plan. The balanced four arc and two arc ASF plans had lower PTV_skin_ *V_95_* coverage by 0.3 pp and 0.9 pp, respectively. The median PTV_skin_ *V*_90_ coverage was higher than 98.6 % in all original and expanded plans. Between the original and 12 mm expanded CTs the median *D_skin_* decreased by 4.0–5.1 pp in the VB and ASF plans. The arc dominant ASF plans had the lowest *D_skin_* in the original and expanded CTs. On the 12 mm expanded CT, CTV_skin_ *V_95_* coverage of nine out of the sixty ASF plans was over 5 pp lower than in the respective VB plan. Five of these nine plans were optimized with the ‘Arc Dominant’ setting and only one was with the two arc setup. On one patient the CTV_skin_ *V_95_* coverage differed by 11.4–17.2 pp on the 12 mm expanded plans compared to the VB plan. The maximum difference between the median *D*_skin_ on the VB and the arc dominant ASF plan was 2.0 pp on the 4 mm expanded CT.

The results for whole breast and breast with nodes cases separately are presented in Supplementary Table S2 and S3.

## 4 Discussion

In this study, we evaluated the performance of a new automated skin flash method, comparing it to the manual virtual bolus method. We assessed both two arc and four arc configurations and simulated breast deformation at 4, 8, and 12 mm expansions. The key findings indicated that the median *V*_95_ and *V*_90_ coverages in both CTV_skin_ and PTV_skin_ as well as *D*_skin_ were close to each other between ASF and VB methods.

Our results align with previous studies, such as those by Nicolini et al. [6], who quantified the robustness of VB planning method and achieved PTV_skin_ *V*_95_ coverage of 92.8 % with a 10 mm expansion. Rossi et al. [18] found that with four different methods tested the mean PTV_skin_ *V*_95_ coverage was 68–92 % with 12 mm expansion. Our findings show that median PTV_skin_ *V*_95_ coverage was 88.6 % [76.5–92.9 %] or above in ASF plans. However, with the 12 mm of simulated swelling, nine out of sixty plans CTV_skin_ *V*_95_ coverages were 5.5–17.2 pp lower than the associated VB plan. The observed reductions in PTV_skin_ and CTV_skin_ coverage in ASF cases may result from angle-dependent bolus density utilized in the new method. In the reference virtual bolus method, the bolus density does not depend on the angle of beam incidence. Possible solutions for this could be using different arc setups and STAMP placements, for example preferring higher weight on tangential STAMPs, as suggested by our results. The use of a separate structure for the skin flash along with slightly higher dose objectives, extent of skin flash and bolus HU value could also affect the coverage and needs to be studied more with the ASF method.

Nicolini et al. [6] reported a mean skin dose of 59 % of the prescribed dose with 10 mm simulated swelling when no skin flash method was used, but with VB method they reported a mean skin dose of 80 %. Rossi et al. [18] reported a 3–7 Gy decrease in the near skin dose, with a total dose of 50 Gy. In our study, median *D*_skin_ of 85.7 % [83.2–87.9 %] or above was achieved with 12 mm simulated swelling with the ASF method.

The study has a few limitations that warrant consideration. First, the use of a single HU value and bolus thickness may limit the generalizability of the results. The justification for this was to allow a direct comparison to Nicolini et al. [6]. Previous studies have explored the virtual bolus method with varying approaches, such as the work by Lizondo et al. [22], which optimized bolus thickness and HU values. Their findings suggested that for a 15 mm PTV_skin_ extension, an HU value of −500 offered the best plan robustness without compromising plan quality. Hubley et al. [20] recommended a HU value of −350 and a PTV_skin_ expansion of 7–10 mm for the ASF method. Rossi et al. [21] tested various virtual PTV_skin_ expansions and bolus thicknesses. They concluded that a 5 mm PTV_skin_ extension with an 8 mm bolus provided the best plan quality. Second, full range of patient-specific factors, such as PTV volume and shape for example, could affect ASF performance and were not explored, suggesting an important area for future research. Finally, the relatively small sample size underscores the need for further studies to assess the ASF performance in larger cohorts and in other clinical settings, such as with other anatomical regions.

In conclusion, the median results of ASF method are close to VB method, with effective skin dose management and high PTV coverage. This positions ASF as a promising method particularly in breast cancer treatment planning. To optimize its performance further, future studies should investigate a wider range of optimization parameters and incorporate more patient-specific factors.

## CRediT authorship contribution statement

**Topi Nykänen:** Conceptualization, Methodology, Formal analysis, Writing – original draft, Writing – review & editing, Investigation, Visualization. **Ville Raatikainen:** Funding acquisition, Conceptualization, Writing – original draft, Writing – review & editing. **Aarno Kärnä:** Conceptualization, Writing – review & editing. **Tuomas Koivumäki:** Conceptualization, Writing – review & editing, Project administration, Supervision.

## Declaration of competing interest

The authors declare the following financial interests/personal relationships which may be considered as potential competing interests: Wellbeing Services County of Central Finland and Hospital Nova of Central Finland has a research collaboration with Varian Medical Systems and Topi Nykänen, Ville Raatikainen, Aarno Kärnä and Tuomas Koivumäki have received honoraria support from Varian Medical Systems.

## References

[1] Seppälä J., Vuolukka K., Virén T., Heikkilä J., Honkanen J.T.J., Pandey A. (2019). Breast deformation during the course of radiotherapy: the need for an additional outer margin. Phys Med.

[2] Defour N., Brun T., Massabeau M., Lanaspeze C., Carillo F., Lacaze T. (2013). “Field in field” for the treatment of breast cancer with lymph nodes: a comparative study with conventional radiotherapy using wedge filters. Phys Med.

[3] Cozzi L., Lohr F., Fogliata A., Franceschini D., De Rose F., Filippi A.R. (2017). Critical appraisal of the role of volumetric modulated arc therapy in the radiation therapy management of breast cancer. Radiat Oncol.

[4] Johansen S., Cozzi L., Olsen D.R. (2009). A planning comparison of dose patterns in organs at risk and predicted risk for radiation induced malignancy in the contralateral breast following radiation therapy of primary breast using conventional, IMRT and Volumetric modulated arc treatment techniques. Acta Oncol.

[5] Popescu C.C., Olivotto I.A., Beckham W.A., Ansbacher W., Zavgorodni S., Shaffer R. (2010). Volumetric modulated arc therapy improves dosimetry and reduces treatment time compared to conventional intensity-modulated radiotherapy for locoregional radiotherapy of left-sided breast cancer and internal mammary nodes. Int J Radiat Oncol.

[6] Nicolini G., Fogliata A., Clivio A., Vanetti E., Cozzi L. (2011). Planning strategies in volumetric modulated arc therapy for breast. Med Phys.

[7] Virén T., Heikkilä J., Myllyoja K., Koskela K., Lahtinen T., Seppälä J. (2015). Tangential volumetric modulated arc therapy technique for left-sided breast cancer radiotherapy. Radiat Oncol.

[8] Boman E., Rossi M., Haltamo M., Skyttä T., Kapanen M. (2016). A new split arc VMAT technique for lymph node positive breast cancer. Phys Med.

[9] Koivumäki T., Clivio A., Doolan P., Essers M., Fusella M., Jäckel M. (2025). The current practice of volumetric modulated arc therapy for breast cancer in Europe – A survey by the EFOMP VMAT breast working group. Phys Med.

[10] Tortorelli G., Di Murro L., Barbarino R., Cicchetti S., di Cristino D., Falco M.D. (2013). Standard or hypofractionated radiotherapy in the postoperative treatment of breast cancer: a retrospective analysis of acute skin toxicity and dose inhomogeneities. BMC Cancer.

[11] Yorke E.D., Jackson A., Rosenzweig K.E., Braban L., Leibel S.A., Ling C.C. (2005). Correlation of dosimetric factors and radiation pneumonitis for non–small-cell lung cancer patients in a recently completed dose escalation study. Int J Radiat Oncol Biol Phys.

[12] Jensen C.A., Roa A.M.A., Johansen M., Lund J.-Å., Frengen J. (2018). Robustness of VMAT and 3DCRT plans toward setup errors in radiation therapy of locally advanced left-sided breast cancer with DIBH. Phys Med.

[13] Osman S.O.S., Hol S., Poortmans P.M., Essers M. (2014). Volumetric modulated arc therapy and breath-hold in image-guided locoregional left-sided breast irradiation. Radiother Oncol.

[14] Koivumäki T., Fogliata A., Zeverino M., Boman E., Sierpowska J., Moeckli R. (2018). Dosimetric evaluation of modern radiation therapy techniques for left breast in deep-inspiration breath-hold. Phys Med.

[15] Zhang Q., Liu J., Ao N., Yu H., Peng Y., Ou L. (2020). Secondary cancer risk after radiation therapy for breast cancer with different radiotherapy techniques. Sci Rep.

[16] Tyran M., Tallet A., Resbeut M., Ferre M., Favrel V., Fau P. (2018). Safety and benefit of using a virtual bolus during treatment planning for breast cancer treated with arc therapy. J Appl Clin Med Phys.

[17] Rossi M., Boman E., Skyttä T., Haltamo M., Laaksomaa M., Kapanen M. (2018). Dosimetric effects of anatomical deformations and positioning errors in VMAT breast radiotherapy. J Appl Clin Med Phys.

[18] Rossi M., Virén T., Heikkilä J., Seppälä J., Boman E. (2021). The robustness of VMAT radiotherapy for breast cancer with tissue deformations. Med Dosim.

[19] Fogliata A., Burger H., Groenewald A., Punt L., Parkes J., Cozzi L. (2024). Intensity modulated therapy for patients with breast cancer. practical guidelines and tips for an effective treatment planning strategy. Adv Radiat Oncol.

[20] Hubley E., Koger B., Li T., Salerno M., Scheuermann R.M., Dong L. (2025). Automatic skin flash optimization in breast and chestwall VMAT with static angle modulated ports: effect of HU and flash margin size on plan quality and robustness. J Appl Clin Med Phys.

[21] Rossi M., Boman E., Kapanen M. (2019). Optimal selection of optimization bolus thickness in planning of VMAT breast radiotherapy treatments. Med Dosim.

[22] Lizondo M., Latorre-Musoll A., Ribas M., Carrasco P., Espinosa N., Coral A. (2019). Pseudo skin flash on VMAT in breast radiotherapy: Optimization of virtual bolus thickness and HU values. Phys Med.

[23] Offersen B.V., Boersma L.J., Kirkove C., Hol S., Aznar M.C., Biete Sola A. (2015). ESTRO consensus guideline on target volume delineation for elective radiation therapy of early stage breast cancer. Radiother Oncol.

