# Supplementary material for: Performance evaluation of a new automated skin flash method for radiotherapy of breast cancer
Source: Phys Imaging Radiat Oncol. 2025 Jul 10;35:100810. doi: 10.1016/j.phro.2025.100810 (PMC12281543; doi:10.1016/j.phro.2025.100810)
Supplement: Supplementary Data 1 [file mmc1.pdf]

Homogenity and conformality of the plans

Plans were evaluated for the dose homogeneity (HI) and Paddick dose conformity index (CI). The HI was calculated as follows:

$$\mathrm{HI} = \frac{D2\%(\mathrm{PTV}) - D98\%(\mathrm{PTV})}{D50(\mathrm{PTV})},$$

where $DX\%_{\mathrm{PTV}}$ (Gy) indicates the dose to X% of the PTV volume, and D50(PTV) (Gy) is the median dose. Smaller HI values indicate better dose homogeneity.

The CI was calculated as:

$$\mathrm{CI} = \frac{V95(\mathrm{PTV})(\mathrm{cc})}{V(\mathrm{PTV})(\mathrm{cc})} \frac{V95(\mathrm{PTV})(\mathrm{cc})}{V95(\mathrm{cc})},$$

where V95(cc) and V95(PTV)(cc) were the whole-body and PTV volumes, respectively, that received at least 95% of the prescribed dose, and V(PTV)(cc) is the PTV volume. Higher CI values indicate better conformity.

The virtual bolus (VB) method resulted in a median CI of 0.88[0.81, 0.91]. The automated skin flash (ASF) plans optimized with the arc dominant setting had a median CI of 0.89[0.83, 0.92]. For the balanced ASF plans the four and two arc achieved median CI of 0.89[0.83, 0.91] and 0.86[0.82, 0.91], respectively. The median HI was 0.11[0.10, 0.12] for the VB plans. The arc dominant ASF plans had an HI 0.11[0.09, 0.13]. The four and two arc balanced ASF plans had an HI of 0.10[0.10, 0.12] and 0.11[0.10, 0.12]. Thus, the plans were evaluated to be clinically comparable.

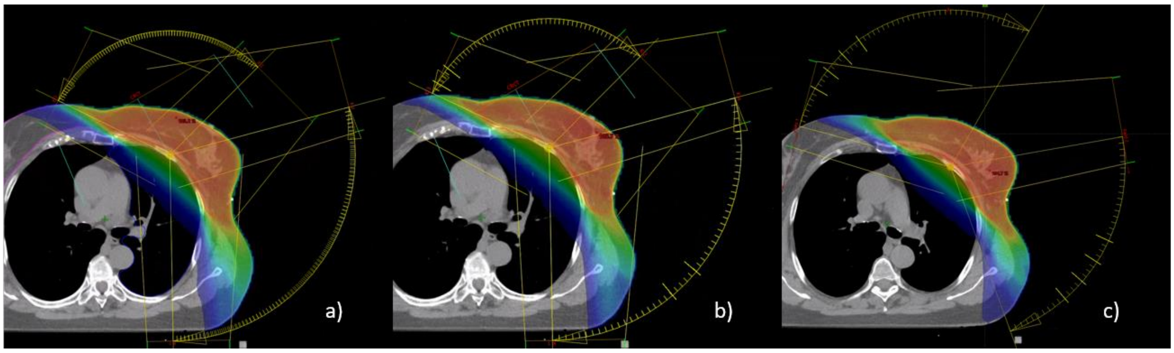

Figure S1 Field setup for the virtual bolus (VB) and the three automatic skin flash (ASF) plans for a whole breast case. Figure shows color wash from dose maximum to 10 % of prescribed dose. a) The four arc VB plan. b) The four arc ASF plans. c) The two arc ASF plan. The dashes along the arc lines illustrate the static angle ports in b) and c).

Dose objectives – left-sided breast treatments

Table S1 Dose objectives used to optimize the breast cancer treatment plans for the study

		Whole breast		Breast with lymph nodes	
**Organ/structure**	**Dose parameter**	**Dose objective**	**Dose constraints**	**Dose objective**	**Dose constraints**
PTV_{skin} (3 mm)	V95%	95%	90%	95%	90%
	V90%	98%	95%	98%	95%
	Dmax		110%		110%
	V107%	< 1 cm^3	< 3 cm^3	< 1 cm^3	< 3 cm^3
Heart	mean	2 Gy	4 Gy	2 Gy	4 Gy
	V16 Gy	2%	5%	5%	15%
	max	< 20 Gy	< 40 Gy	< 20 Gy	< 40 Gy
LAD	V30 Gy	0%	2 %	0%	2%
	V20 Gy	1%	2%	1%	
	mean	5 Gy	10 Gy	5 Gy	10 Gy
Ipsilateral lung	mean	8 Gy	13 Gy	9 Gy	13 Gy
	V16 Gy	15%	20%	20%	35%
	V4 Gy	35%	50%	50%	65%
Contralateral lung	mean	1 Gy	2.5 Gy	1.5 Gy	2.5 Gy
Spinal cord	Dmax			15 Gy	38 Gy
Contralateral breast	mean	1 Gy	3 Gy	3 Gy	7 Gy

Whole breast and breast with nodes result tables

Table S2 The median of PTV coverages and mean skin doses for whole breast virtual bolus (VB) and automatic skin flash (ASF) treatment plans. Minimum and maximum values are presented in square brackets. (AD=Arc Dominant, B=Balanced)

	CTV_{Skin}		PTV_{skin}		Skin
	V_{95} (%)	V_{90} (%)	V_{95} (%)	V_{90} (%)	$\underline{D_{skin}}$ (%)
			Original		
**4 ARC VB**	98.3 [97.1, 98.8]	99.9 [99.5, 100.0]	96.1 [95.1, 97.4]	99.7 [99.3, 99.8]	91.4 [89.7, 92.4]
**4 ARC AD ASF**	96.9 [95.5, 98.7]†	99.6 [98.8, 100.0]	95.8 [95.0, 97.1]	99.4 [98.7, 100.0]	90.1 [88.5, 91.1]†
**4 ARC B ASF**	97.7 [96.0, 98.3]*	99.9 [99.5, 100.0]	95.9 [95.0, 96.6]	99.7 [99.3, 99.9]	90.5 [88.8, 91.6]*
**2 ARC B ASF**	97.7 [96.5, 98.4]*	99.9 [99.6, 100.0]‡**	95.5 [95.1, 95.8]*	99.6 [99.4, 99.8]	90.8 [89.5, 91.4]‡
			Expansion 4 mm		
**4 ARC VB**	96.1 [94.9, 97.0]	99.7 [98.9, 99.8]	94.3 [92.0, 95.8]	99.4 [98.7, 99.7]	88.8 [86.4, 90.0]*
**4 ARC AD ASF**	94.4 [92.4 95.6]†	98.7 [97.9, 99.8]†	93.2 [91.8, 95.2]	98.9 [97.7, 99.7]†	87.1 [85.0, 88.3]†
**4 ARC B ASF**	94.8 [93.4, 96.5]‡*	99.3 [98.5, 99.7]*	93.5 [92.3, 95.3]	99.1 [98.3, 99.7]	87.5 [84.9, 89.0]*
**2 ARC B ASF**	95.2 [94.1, 96.3]‡*	99.4 [98.6, 99.8]‡**	93.3 [92.7, 94.3]*	99.3 [98.6, 99.5]‡	87.8 [86.0, 89.4]‡
			Expansion 8 mm		
**4 ARC VB**	95.4 [93.2, 96.2]	99.5 [98.5, 99.8]	93.1 [89.1, 94.0]	99.0 [98.4, 99.5]	87.2 [85.4, 89.2]
**4 ARC AD ASF**	92.3 [89.2, 95.1]†	98.5 [97.2, 99.7]†	91.3 [88.1, 93.4]†	98.6 [97.5, 99.5]†	86.1 [83.7, 87.8]†
**4 ARC B ASF**	93.1 [89.7, 95.9]*	99.0 [97.8, 99.7]‡*	91.7 [89.3, 93.8]	98.8 [98.2, 99.5]	86.4 [83.9, 88.5]*
**2 ARC B ASF**	93.3 [91.7, 95.2]‡*	99.1 [98.3, 99.7]‡	91.5 [90.0, 93.0]*	98.9 [98.4, 99.2]	86.7 [84.2, 87.8]‡*
			Expansion 12 mm		
**4 ARC VB**	92.5 [89.9, 94.3]	99.1 [98.5, 99.4]	90.2 [85.8, 92.2]	98.9 [98.2, 99.3]	86.2 [85.5, 88.0]
**4 ARC AD ASF**	89.5 [84.9, 93.6]†	98.5 [97.6, 99.5]	88.2 [83.8, 92.9]	98.7 [97.6, 99.6]	85.7 [83.2, 87.9]
**4 ARC B ASF**	90.6 [89.7, 94.6]	98.8 [97.4, 99.7]	89.6 [85.3, 93.5]	98.7 [97.8, 99.6]	86.3 [82.8, 87.8]
**2 ARC B ASF**	90.3 [88.3, 92.4]*	98.8 [98.2, 99.3]	88.4 [85.4, 90.5]*	98.6 [98.4, 99.2]	86.1 [84.7, 86.8]*

†Denotes statistically significant difference between VB and arc dominant four arc ASF ($p<0.05$)

‡ Denotes statistically significant difference between arc dominant four arc ASF and balanced four arc ASF or balanced two arc ASF ($p<0.05$)

*Denotes statistically significant difference between VB and balanced four arc ASF or balanced two arc ASF ($p<0.05$)

**Denotes statistically significant difference between balanced four arc ASF and balanced two arc ASF ($p<0.05$)

Table S3 The median of PTV coverages and median skin doses for breast with nodes virtual bolus (VB) and automatic skin flash (ASF) treatment plans. Minimum and maximum values are presented in square brackets. (AD=Arc Dominant, B=Balanced)

	CTV_{Skin}		PTV_{skin}		Skin
	V_{95} (%)	V_{90} (%)	V_{95} (%)	V_{90} (%)	D_{skin} (%)
			Original		
**4 ARC VB**	98.6 [96.8, 99.4]	100.0 [99.8, 100.0]	95.6 [95.0, 96.6]	99.8 [99.6, 99.9]	91.0 [89.4, 93.0]
**4 ARC AD ASF**	97.9 [95.9, 98.7]†	99.9 [99.4, 100.0]†	95.7 [95.0, 96.7]	99.6 [99.2, 99.8]†	89.4 [84.9, 91.2]†
**4 ARC B ASF**	98.5 [96.3, 99.3]‡	100.0 [99.6, 100.0]‡	96.1 [95.3, 97.3]	99.6 [99.3, 99.9]	90.4 [89.3, 93.0]‡
**2 ARC B ASF**	98.7 [95.9, 99.3]‡	99.9 [99.8, 100.0]‡	96.2 [95.1, 97.0]	99.7 [99.3, 99.9]*	90.6 [89.3, 93.4]‡
			Expansion 4 mm		
**4 ARC VB**	96.9 [95.9, 98.6]	99.8 [99.5, 100.0]	93.9 [93.5, 95.1]	99.5 [99.3, 99.8]	88.5 [86.8, 90.8]
**4 ARC AD ASF**	95.2 [90.0, 97.0]†	99.4 [97.1, 99.9]†	93.6 [90.8, 94.7]†	99.2 [97.9, 99.6]†	86.7 [84.6, 88.7]†
**4 ARC B ASF**	95.9 [92.7, 98.4]‡	99.6 [99.1, 100.0]‡	93.8 [92.5, 95.9]‡	99.3 [98.9, 99.8]*	87.3 [85.9, 90.4]‡
**2 ARC B ASF**	96.9 [93.5, 98.2]‡	99.7 [99.2, 100.0]‡	94.2 [92.5, 96.4]‡	99.4 [99.0, 99.8]*‡	87.5 [86.3, 91.4]‡
			Expansion 8 mm		
**4 ARC VB**	96.0 [93.9, 97.5]	99.7 [99.2, 99.9]	91.9 [90.9, 94.0]	99.2 [98.9, 99.7]	87.2 [85.5, 89.6]
**4 ARC AD ASF**	94.4 [84.9, 96.4]†	99.3 [95.9, 99.8]†	91.9 [84.9, 94.1]	99.0 [96.5, 99.5]†	85.7 [83.5, 88.2]†
**4 ARC B ASF**	94.7 [88.0, 98.1]‡	99.4 [97.4, 99.9]	91.4 [87.2, 95.3]	98.8 [97.7, 99.7]	86.3 [85.0, 89.8]‡
**2 ARC B ASF**	95.6 [89.6, 98.5]‡	99.6 [98.2, 100.0]‡	92.5 [87.9, 96.4]‡	99.1 [98.0, 99.8]**‡	86.6 [85.4, 91.1]‡
			Expansion 12 mm		
**4 ARC VB**	92.3 [89.4, 95.3]	98.9 [98.3, 99.5]	89.1 [86.8, 92.0]	98.7 [98.3, 99.4]	85.9 [84.1, 87.7]
**4 ARC AD ASF**	91.3 [76.9, 95.0]	99.0 [96.6, 99.6]	89.0 [76.5, 92.5]	98.6 [96.6, 99.5]	85.7 [84.3, 87.9]
**4 ARC B ASF**	91.2 [80.2, 97.1]‡	99.1 [98.1, 99.9]‡	89.1 [80.2, 95.0]	98.8 [97.9, 99.8]	86.4 [84.7, 90.1]‡
**2 ARC B ASF**	92.8 [82.7, 97.9]	99.3 [98.0, 99.9]‡*	90.0 [81.9, 96.5]	98.9 [97.6, 99.8]‡	86.5 [85.1, 90.9]‡

†Denotes statistically significant difference between VB and arc dominant four arc ASF ($p<0.05$)

‡ Denotes statistically significant difference between arc dominant four arc ASF and balanced four arc ASF or balanced two arc ASF ($p<0.05$)

*Denotes statistically significant difference between VB and four arc ASF or two arc ASF ($p<0.05$)

**Denotes statistically significant difference between four arc ASF and two arc ASF ($p<0.05$)
